# Extracellular Enzyme Activity Profile in a Chemically Enhanced Water Accommodated Fraction of Surrogate Oil: Toward Understanding Microbial Activities After the Deepwater Horizon Oil Spill

**DOI:** 10.3389/fmicb.2018.00798

**Published:** 2018-04-24

**Authors:** Manoj Kamalanathan, Chen Xu, Kathy Schwehr, Laura Bretherton, Morgan Beaver, Shawn M. Doyle, Jennifer Genzer, Jessica Hillhouse, Jason B. Sylvan, Peter Santschi, Antonietta Quigg

**Affiliations:** ^1^Department of Marine Biology, Texas A&M University at Galveston, Galveston, TX, United States; ^2^Department of Marine Science, Texas A&M University at Galveston, Galveston, TX, United States; ^3^Department of Oceanography, Texas A&M University, College Station, TX, United States

**Keywords:** enzymes, aggregates, EPS, oil, corexit, bacteria

## Abstract

Extracellular enzymes and extracellular polymeric substances (EPS) play a key role in overall microbial activity, growth and survival in the ocean. EPS, being amphiphilic in nature, can act as biological surfactant in an oil spill situation. Extracellular enzymes help microbes to digest and utilize fractions of organic matter, including EPS, which can stimulate growth and enhance microbial activity. These natural processes might have been altered during the 2010 Deepwater Horizon oil spill due to the presence of hydrocarbon and dispersant. This study aims to investigate the role of bacterial extracellular enzymes during exposure to hydrocarbons and dispersant. Mesocosm studies were conducted using a water accommodated fraction of oil mixed with the chemical dispersant, Corexit (CEWAF) in seawater collected from two different locations in the Gulf of Mexico and corresponding controls (no additions). Activities of five extracellular enzymes typically found in the EPS secreted by the microbial community – α- and β-glucosidase, lipase, alkaline phosphatase, leucine amino-peptidase – were measured using fluorogenic substrates in three different layers of the mesocosm tanks (surface, water column and bottom). Enhanced EPS production and extracellular enzyme activities were observed in the CEWAF treatment compared to the Control. Higher bacterial and micro-aggregate counts were also observed in the CEWAF treatment compared to Controls. Bacterial genera in the order *Alteromonadaceae* were the most abundant bacterial 16S rRNA amplicons recovered. Genomes of *Alteromonadaceae* commonly have alkaline phosphatase and leucine aminopeptidase, therefore they may contribute significantly to the measured enzyme activities. Only *Alteromonadaceae* and *Pseudomonadaceae* among bacteria detected here have higher percentage of genes for lipase. *Piscirickettsiaceae* was abundant; genomes from this order commonly have genes for leucine aminopeptidase. Overall, this study provides insights into the alteration to the microbial processes such as EPS and extracellular enzyme production, and to the microbial community, when exposed to the mixture of oil and dispersant.

## Introduction

The Deepwater Horizon (DwH) oil spill in the Gulf of Mexico was a catastrophic event that released 4.9 million barrels of oil (Turner et al., 2014). This was followed by the use of ∼2.9 million liters of the dispersing agent Corexit in an attempt to clear the oil from the surface of the ocean and protect coastal ecosystems (Kujawinski et al., 2011). Months after this event, the oil disappeared from the surface ocean, with factors such as natural light induced photo-oxidation, volatilization, sedimentation, and microbial degradation playing an important role (Mendelssohn et al., 2012). The ability of some bacterial taxa to degrade oil has been well established (Bailey et al., 1973), and oil biodegradation during the DwH spill has been extensively reported (Hazen et al., 2010; Valentine et al., 2010; Kostka et al., 2011). Following the oil spill, extracellular polymeric substances (EPS) combined with particulates and other materials, operationally defined as marine snow (Quigg et al., 2016), were observed in large amounts (Passow et al., 2012). Much of this marine snow was also found to have oil included in the aggregated material, so that it was referred to as ‘marine oil snow’ (MOS) by Passow et al. (2012). Enhanced EPS production in the form of transparent exopolymer particles (TEP) was observed in the presence of oil; this material might have acted as a bio surfactant (Kleindienst et al., 2015b).

Given that EPS production by phytoplankton can vary from 3 to 40% of the total primary productivity (Engel, 2002), and considering primary productivity accounts for 45–50 Pg C yr^-1^ in the ocean (Longhurst et al., 1995; Field et al., 1998), the amount of carbon released as EPS could range between 1.5 and 20 Pg C yr^-1^. Furthermore, bacteria are also known to produce EPS (Manivasagan and Kim, 2014), and these EPS have been implicated to play a protective role against adverse environmental condition (Poli et al., 2010). EPS is heterogeneous in composition, consisting mainly of carbohydrates, proteins, monomers of sugars, amino acids, and uronic acids (Underwood et al., 2004; Xu et al., 2011a,b; Quigg et al., 2016). EPS can act as a carbon, nitrogen and phosphorus substrate that assists the growth of bacteria and mixotrophic phytoplankton, thereby boosting microbial activity (Decho, 1990). Several studies have shown that extracellular enzymes facilitate breakdown of complex polymers in EPS to less complex molecules (Jones and Lock, 1993; Romaní and Sabater, 2000; Espeland et al., 2001), which heterotrophic microbes would otherwise not be able to use. Heterotrophic microbes secrete several types of extracellular enzymes (exoenzymes) that assist them in degrading the various components of EPS (Decho, 1990) such as α- and β-glucosidase, alkaline phosphatase, leucine amino-peptidase, and lipase (Yamada and Suzumura, 2010). As enzymes are specific to the substrates they act upon and microbes vary in their ability to produce different kinds of enzymes, the differences in activities between enzymes can be used as an indirect indicator of microbial functional diversity in the system and/or the nutrient composition of the system (Caldwell, 2005).

In addition to the EPS, the oil and Corexit can act as a source of carbon to the microbes during the DwH oil spill, which may have affected EPS production and enzyme activities. Oil and Corexit has also known to cause a significant change in the microbial community, favoring hydrocarbon degraders (Bacosa et al., 2015b; Kleindienst et al., 2015a; Doyle et al., 2018). Therefore, how the addition of oil and Corexit can affect EPS production, extracellular enzyme production, microbial community and aggregate formation needs to be examined. In this study, we conducted mesocosm scale experiments with seawater from two sites in the Gulf of Mexico. A chemically enhanced water accommodated fraction of oil (CEWAF) prepared from a mixture of oil and Corexit (ratio of 20:1) in seawater and Controls (no additions) were used to test the effects of oil/dispersant mixture on the microbial community, their enzymatic activity and EPS production in three different layers within the mesocosm tanks (surface, water column, and bottom).

## Materials and Methods

### Mesocosm Study

Two mesocosm studies were carried out using seawater collected from the Gulf of Mexico during July 2016. The first site located at 27°53N, 94°2W (salinity: 30.77 ppt, pH: 8.38, temperature: 30.8°C) was chosen as an offshore, open ocean site (∼174 km from shore) while the second site at 29°22N, 93°23W (salinity: 31.13 ppt, pH: 8.02, temperature: 30.5°C) was chosen as a coastal site (∼20 km from shore). These will be referred to as offshore and coastal respectively. The seawater was supplemented with nutrients at f/20 concentrations (Guillard and Ryther, 1962) before starting the six mesocosms (3 controls, 3 CEWAF). The seawater was used directly as a Control treatment (87 L each mesocosm). Macondo surrogate oil (25 ml) and dispersant in the ratio of 20:1 were combined to produce a chemically enhanced water accommodated fraction (CEWAF) of oil according to Wade et al. (2017). Briefly, the oil and Corexit were added together before being transferred to the corresponding seawater and mixed in 130 L circulating baffled tanks for 24 h under low light and ambient temperature (∼21°C). At the end of this period, the 87 L of CEWAF was transferred to the mesocosm tanks by pumping from the bottom of the baffled tank in order to avoid the surface slick. The initial nitrate and phosphate concentration were 97 (±4.7) μMol.L^-1^ and 11.2 (±0.3) μMol.L^-1^ in the Control and 119 (±1.9) μMol.L^-1^ and 5.5 (±0.4) μMol.L^-1^ in the CEWAF of the offshore mesocosm. Whereas, the initial nitrate and phosphate concentration were 133.3 (±33.8) μMol.L^-1^ and 10.2 (±0.2) μMol.L^-1^ in the Control and 130.0 (±7.4) μMol.L^-1^ and 11.0 (±0.3) μMol.L^-1^ in the CEWAF of the coastal mesocosm respectively. The initial estimated oil equivalent (EOE) concentration in the offshore and coastal CEWAF treatment were 39.06 (±0.77) mg.L^-1^ and 81.06 (±20.50) mg.L^-1^, respectively. The incubation time for the offshore and coastal mesocosms were 96 hrs and 72 h respectively. The incubation time for both the mesocosm were decided based on the percentage of oil consumed/remaining in the CEWAF tanks. Due to technical issues associated with replicating CEWAF with the same initial oil concentration, the percentage oil concentration was chosen as the deciding parameter over actual oil concentration in terminating the study. The offshore mesocosms took 96 h to reach ∼20% of the initial oil concentration, whereas it only took 72 h in the coastal mesocosm.

### Sample Collection

Samples for enzyme activity, EPS composition, and total organic carbon (TOC), dissolved organic carbon (DOC), and particulate organic carbon (POC) analysis were collected from three different layers of the mesocosm tanks on the last day. The mesocosm tanks were 74.5 cm long and 43 cm wide. Samples collected in the top 2–5 cm of the tanks were designated as the surface. Samples collected through a spigot mounted on the side of the tanks were designated as water column. Finally, samples collected from the floor of the tanks were designated as the bottom. A syringe was used to collect the samples from the surface and bottom respectively. The samples were collected from these three layers in order to account for the differences in aggregation (higher in the bottom layer for Control and in the surface for CEWAF treatment) observed during the experiment. Samples for bacterial community composition and micro-aggregate counts/sizes were collected concurrently from the water column layer.

### Enzyme Assays

Enzyme activities for α- and β-glucosidase, alkaline phosphatase, leucine amino-peptidase, and lipase were measured on the last day according to Yamada and Suzumura (2010). The enzymes and the substrates used in this study are listed in **Table 1**. The substrates were dissolved in milli-Q water so that the final stock solutions were 1 mM. The substrate for 4-Methylumbelliferyl oleate was dissolved in minute volume (250 μl) of DMSO and the concentration was adjusted with milli-Q water to a final concentration of 1 mM, Substrates were then added to Control and CEWAF samples in triplicate to a final concentration of 0.2 mM. The samples were then incubated at room temperature in the dark for 3 h. After incubation, the reactions were stopped by the addition of 1 mL of borate buffer solution (0.4 M) adjusted to pH 8.0 for 7-amido-4-methylcoumarin (AMC)-tagged substrates or pH 10.0 for 4-methylumbelliferyl (MUF)-tagged substrates. The fluorescence intensity was then measured at excitation/emission wavelengths (nm) of 380/440 (AMC) or 365/448 (MUF) using a spectrofluorometer (Shimadzu RF-5300). The measurements were then corrected with the blank values obtained using heated seawater samples (80°C for 15 min) in duplicate at the beginning of the incubation. Respective substrates corresponding to the different enzymes were added to the blank samples prior to incubation and measurement.

**Table 1:** List of enzymes with fluorescent substrate used in this study.

Enzyme	Substrate	Catalog number
α-*Glucosidase*	4-Methylumbelliferyl α-D-glucopyranoside	M9766 (Sigma-Aldrich)
β-*Glucosidase*	4-Methylumbelliferyl β-D-glucopyranoside	M3633 (Sigma-Aldrich)
*Lipase*	4-Methylumbelliferyl oleate	M2639 (Sigma-Aldrich)
*Alkaline phosphatase (AP)*	4-Methylumbelliferyl phosphate	M8883 (Sigma-Aldrich)
*Leucine amino-peptidase (LAP)*	Leu-AMC hydrochloride	ab145346 (Abcam)


### EPS Analysis

Extracellular polymeric substances composition was measured in terms of carbohydrate, protein, and uronic acid content and total EPS was calculated by summing these parameters. Particles were collected with a polycarbonate filter (0.4 μm, Millipore, United States), and the attached EPS from the particles was then extracted with 0.35 M EDTA followed by an ultrafiltration step to remove the salts and excessive EDTA (Xu et al., 2009, 2011a,b). EPS from the dissolved phase was directly obtained by concentrating and desalting using an Amicon Ultra-15 centrifugal filter unit with ultracel-3 membrane (Millipore, 3 kDa). The carbohydrate concentration in the EPS was determined by anthrone method with glucose as the standard (Yemm and Willis, 1954). The protein content of EPS was determined with the help of a Pierce BCA protein assay kit based on a modified bicinchoninic acid method with bovine serum albumin as the standard (Smith et al., 1985). Uronic acids in the EPS were estimated by the addition of sodium borate (75 mM) in concentrated sulfuric acid and *m-*hydroxydiphenyl according to Blumenkrantz and Asboe-Hansen (1973) with glucuronic acid as the standard for this assay.

### TOC, DOC, and POC Analysis

TOC and DOC were determined using a Shimadzu TOC-L analyzer (Xu et al., 2011b). For POC analysis, water sample was filtered through a pre-combusted GF/F membrane (0.7 μm, Whatman, United States), and then quantified using a Perkin Elmer Series II CHNS 2400 analyzer, after HCl-fuming to remove the carbonates. Acetanilide (71.09%) was used as the analytical standard (Xu et al., 2011a). Samples from the offshore mesocosm were limited, therefore only samples from the coastal mesocosm were analyzed.

### Dissolved Oxygen (DO) Concentration

A calibrated 556 MPS YSI meter (Yellow Springs, OH, United States) fitted with a DO/Temperature sensor (5563-10) was used to measure the DO (mg L^-1^) directly in the surface and bottom of each mesocosm tank on the last day of each mesocosm experiment.

### Microbial and Micro-Aggregate Counts/Sizes

Direct cell counts were performed on the samples collected from the water column on the last day in three replicate tanks per treatment. Samples were visualized with an epifluorescence microscope (Zeiss Axio Imager.M2) after staining the fixed samples with DAPI (45 μM final concentration) for 5 min in the dark and filtering them onto 25 mm, 0.2 μm black polycarbonate filters, according to Doyle et al. (2018). Microbial cell counts were performed at 1000× magnification and, due to their much larger size, micro-aggregates were quantified at 400× magnification. For micro-aggregate abundance, the presence of a micro-aggregate was counted, not the number of cells present per micro-aggregate. Micro-aggregates were defined as groups of cells in clumps 10–200 μm in diameter, often found gathered around drops of oil.

Size fraction analysis of aggregates was also performed using Z1 dual-threshold Coulter counter (Beckman Coulter). It should be noted that these aggregates do not exactly correspond to the microbial micro-aggregates measured above, and likely include those as well as other particles in that size fraction. Samples (15 mL) from the water column were taken on the last day and analyzed immediately. Particles of four different size ranges (5–10, 10–20, 20–50, and >50 μm) were counted with a 100 μm aperture. A sample of filtered seawater was used as blank (typically less than 10 particles were counted). Samples were diluted with filtered seawater (0.2 μm) if the particle coincidence at the aperture exceeded 5%, where particle coincidence is the chance of more than one particle passing through the aperture at once.

### Bacterial Community Composition

Prokaryotic community composition (Bacteria and Archaea) was analyzed as described in detail in Doyle et al. (2018). Briefly, samples (150 ml) collected from the water column in three replicate tanks per treatment concurrently with other samples were pre-filtered through 10 μm filters to remove most eukaryotic cells followed by filtration onto 47 mm 0.22 μm Supor PES filter membranes (Pall). Total DNA was extracted from filters using FastDNA Spin kits (MP Biomedical). 16S rRNA gene (hyper-variable V4 region) was PCR amplified with GoTaq Flexi DNA Polymerase (Promega) according to Caporaso et al. (2012), with specifics in Doyle et al. (2018). Amplifications were performed using the 515F-806R universal primer pair according to recent revisions (Apprill et al., 2015; Parada et al., 2016), which included Golay barcodes and adapters for Illumina MiSeq sequencing. The products were combined and quantified with the QuantiFluor dsDNA System (Promega), pooled and purified with an UltraClean PCR Clean-Up Kit (MoBio Laboratories). The library, along with the three sequencing primers, were sent to the Georgia Genomics Facility (Athens, GA, United States) for MiSeq sequencing (v2 chemistry, 2 × 250 bp). Sequence processing was carried out using mothur v.1.36.1 (Schloss et al., 2009) following a modified version of the protocol described in Kozich et al. (2013). Analysis of rarefaction curves was conducted using all available reads. Generation of operational taxonomic units (OTUs) and analysis of alpha and beta diversity was conducted using a dataset subsampled to 39,054 samples per read (Supplementary Table S1). Goods coverage was >0.99 for all samples.

Raw DNA sequence data used in this project can be found in the NCBI Genbank database under accession numbers SAMN07795505-SAMN07795510 (Offshore) and SRR6176504, SRR6176505, SRR6176497, SRR6176488, SRR6176473 and SRR6176442 (Coastal).

### Screening of Microbial Genomes

To determine which of the most relatively abundant bacterial families detected are potentially capable of extracellular enzyme production, we searched the Integrated Microbial Genomes (IMG) database (Markowitz et al., 2006) for genomes within these orders using the “Find Functions” search and the “Enzymes (list)” filters for each bacterial order searched, similar to previous work (Jacobson Meyers et al., 2014). EC 3.4.11.1 was used to search for the gene(s) encoding leucine aminopeptidase, EC 3.1.3.1 was used to search for the gene(s) encoding alkaline phosphatase, EC 3.1.1.3 was used to search for genes encoding for lipase, EC 3.2.1.20 was used to search for genes encoding α-glucosidase, and EC 3.2.1.21 was used to search for genes encoding β-glucosidase. Genomes were scored as positive if they contained a gene that encoded for an exoenzyme, and then the number of genes within the family was tallied to calculate the percentage of genomes within that family capable of producing each enzyme. This analysis depends on a few assumptions: (a) if an organism is a member of a clade in which a specific enzyme is more abundant, then that organism is more likely to have the enzyme, (b) the annotations in IMG are reliable, and (c) that the exoenzymes assayed are expressed extracellularly, rather than intracellularly.

### Statistical Analysis

The results were statistically analyzed by using one-way ANOVA with multiple comparisons of the mean of each group with the mean of every other group using a Tukey test. These statistical analyses were performed using GraphPad Prism software (version 7.0f).

## Results

**FIGURE 1:**
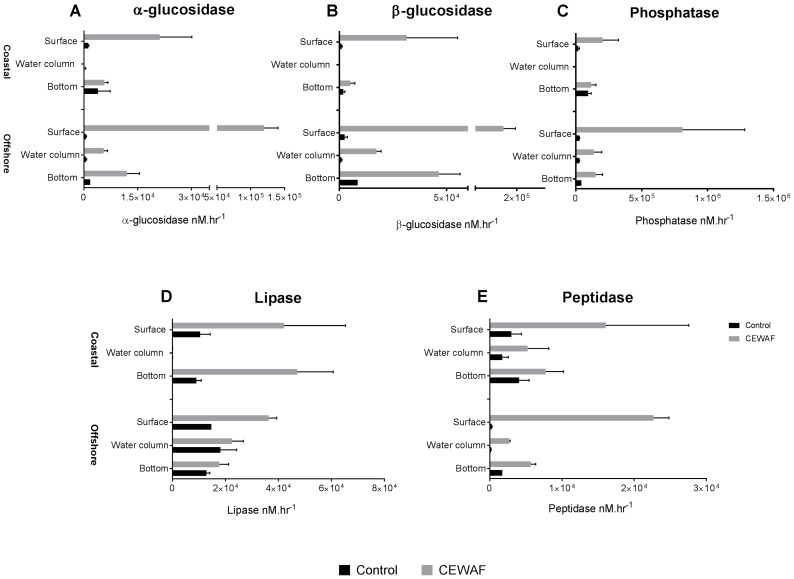
Average enzyme activities (±SD) of **(A–E)** α- and β-glucosidase, alkaline phosphatase, lipase, leucine amino-peptidase measured in the surface, water column and bottom of the offshore and coastal mesocosms.

Measurement of α-glucosidase activities in different layers of the CEWAF tanks revealed highest activity at the surface in both the offshore and the coastal mesocosms (One way ANOVA: *p* < 0.02; **Figure 1A**). Similar patterns were seen for both β-glucosidase and alkaline phosphatase, although the differences in alkaline phosphatase were not statistically significant (**Figures 1B,C**). In the Control tanks, the activities of α-glucosidase, β-glucosidase and alkaline phosphatase were significantly higher at the bottom than in other layers in the offshore mesocosm (One way ANOVA: *p* < 0.0005; **Figure 1**). While the activities were similarly higher at the bottom layer in the Control tanks of the coastal mesocosm, the differences were statistically significant only for alkaline phosphatase (One way ANOVA: *p* < 0.0015). Comparison of Control vs. CEWAF tanks in the coastal mesocosm revealed significant differences for α-glucosidase, β-glucosidase and alkaline phosphatase only at the surface (Two way ANOVA: *p* < 0.004). However, for the offshore mesocosm, α-glucosidase and β-glucosidase activities were significantly higher in CEWAF compared to Control in all three layers (Two way ANOVA: *p* < 0.0001), whereas alkaline phosphatase was significantly higher only at the surface (Two way ANOVA: *p* = 0.0011).

Measurement of lipase activities revealed slightly different profile compared to α-glucosidase, β-glucosidase and alkaline phosphatase (**Figure 1D**). For CEWAF tanks, lipase activities were highest in the surface compared to other layers in the offshore mesocosm (Two way ANOVA: *p* < 0.002), similar to that observed for α-glucosidase, β-glucosidase and alkaline phosphatase. However, in the coastal mesocosm, lipase activities were the lowest in the water column (Two way ANOVA: *p* < 0.002), and similar between surface and bottom layer in CEWAF tanks. Comparison of Control vs. CEWAF tanks in the offshore mesocosm revealed significant differences only at the surface for the offshore mesocosm (Two way ANOVA: *p* < 0.0001). However, in the coastal mesocosm, lipase activities were significantly higher in CEWAF than Control in both surface and bottom layers (Two way ANOVA: *p* < 0.004).

Measurement of leucine aminopeptidase activities in different layers of the CEWAF tanks revealed highest activity at the surface in the offshore mesocosm (One way ANOVA: *p* < 0.0001; **Figure 1E**). A similar pattern was observed for leucine aminopeptidase in the coastal mesocosm, however the differences were not statistically significant (**Figure 1**). In Control tanks, the activities were higher at the bottom for leucine aminopeptidase in the offshore mesocosm (One way ANOVA: *p* < 0.0001), whereas the activities were similar in the coastal mesocosm. CEWAF had significantly higher leucine aminopeptidase activities than the Control in all the layers for the offshore mesocosm (Two way ANOVA: *p* < 0.02), however, significant differences were only seen at the surface for the coastal mesocosm (Two way ANOVA: *p* = 0.02). Overall, most of the enzymes showed higher activities at the surface for CEWAF treatments and at the bottom for Control treatment in the offshore and/ or coastal mesocosms (**Figure 1**). In addition, overall enzyme activities were significantly higher in CEWAF treatment than in the Control for both offshore (Unpaired *t*-test: *p* = 0.0057) and coastal (Unpaired *t*-test: *p* = 0.0312) experiment (Supplementary Figure S1).

### EPS Composition Across the Layers

**FIGURE 2:**
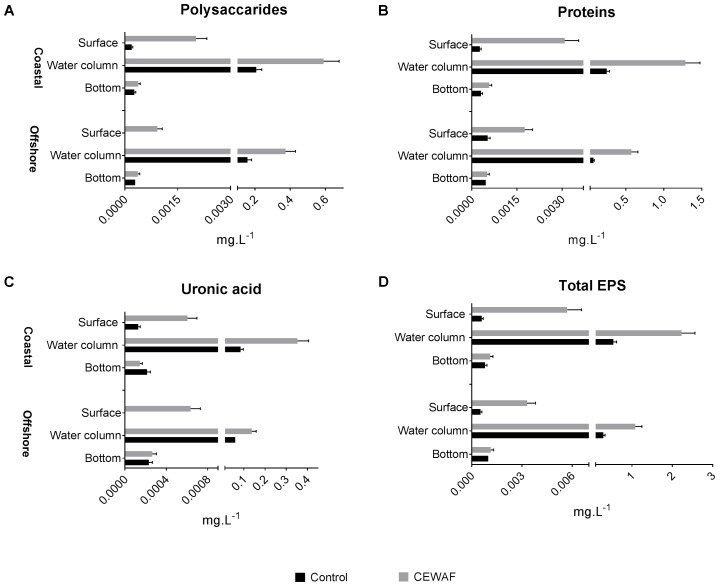
**(A–D)** Average polysaccharide, protein and uronic acid content (±SD) of EPS at measured in the surface, water column and bottom layers of offshore and coastal mesocosms.

The polysaccharide, protein, and uronic acid content was measured to determine the overall EPS composition (**Figure 2**). Total EPS concentration was highest in the water column in both treatments for both mesocosms (Two-way ANOVA: *p* < 0.0001). In Control tanks, significantly higher concentration of EPS was observed at the bottom layer compared to the surface layer in the offshore mesocosm (Unpaired *t*-test: *p* = 0.009). Similar trends were observed in the coastal mesocosm samples, however, the differences were not statistically significant (**Figure 2D**). In the CEWAF tanks, there was more EPS in the surface than the bottom layer in both the mesocosms (Unpaired *t*-test: *p* < 0.003). Exposure to CEWAF treatment significantly increased the amount of polysaccharide, proteins and uronic acids produced in the water column and in the surface (Two-way ANOVA: *p* < 0.0001; **Figure 2**). Comparison of EPS composition in the water column of CEWAF relative to the Control, showed higher production of proteins in both the coastal (5.3 fold) and offshore (8.1 fold) mesocosms followed by uronic acids (4.4 and 2.6 fold) and carbohydrates (2.9 and 2.4 fold). Comparison of EPS composition in the surface of CEWAF relative to the Control showed similar patterns to that observed in the water column.

### TOC, DOC, and POC

**FIGURE 3:**
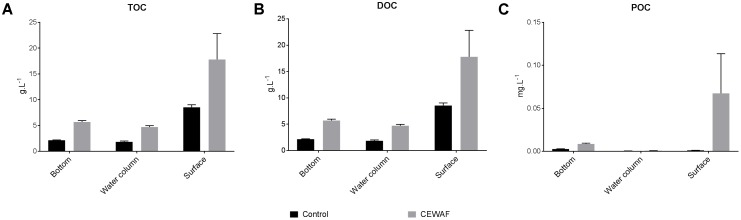
**(A–C)** Average TOC, DOC, and POC levels (±SE) of water samples from surface, water column and the bottom of the coastal mesocosms.

In the coastal mesocosm, TOC and DOC concentrations were highest in the surface layers in both treatments (Two-way ANOVA: *p* < 0.0025) (**Figure 3A**). Overall, there was significantly more TOC and DOC in the CEWAF tanks than in the Control tanks at all layers (**Figure 3B**) (Two-way ANOVA: *p* = 0.0007). POC concentrations were significantly higher in the CEWAF treatment in the surface layer than in the Control (Unpaired *t*-test: *p* < 0.00001) (**Figure 3C**). The bottom POC concentration was higher than both the water column (Two-way ANOVA: *p* = 0.0007) and surface layers (Two-way ANOVA: *p* = 0.0056) in the Control treatment. In the CEWAF treatment, the POC was higher at the surface than in the water column and bottom (**Figure 3C**), although these differences were not statistically significant (One-way ANOVA: *p* > 0.2571).

### Dissolved Oxygen Concentration

The DO concentration in the CEWAF mesocosms was very low on the last day, decreasing to almost zero at the bottom of the offshore and coastal tanks (**Table 2**). A decrease in DO in the CEWAF treatments from 0.3 (±0.12) mg L^-1^ at the surface to 0.1 (±0.04) mg L^-1^ at the bottom of the tanks was observed in offshore and 0.4 (±0.01) mg L^-1^ to 0.22 (±0.04) mg L^-1^ in coastal tanks (**Table 2**). These concentrations are considered to be hypoxic to anoxic. By comparison, the Control treatments had a significantly higher dissolved oxygen concentration throughout the tank with an average value of 6 (±0.37) mg L^-1^ in offshore and 7.5 (±1.11) mg L^-1^ in the coastal mesocosms from surface to bottom.

**Table 2:** Average dissolved oxygen concentration (±SD) at the surface and bottom in control and CEWAF treatments of the offshore and coastal mesocosm tanks.

	Control		CEWAF	
		
	Surface mg L^-1^	Bottom mg L^-1^	Surface mg L^-1^	Bottom mg L^-1^
Offshore	6.31 (±0.06)	5.67 (±0.65)	0.33 (±0.12)	0.16 (±0.04)
Coastal	7.54 (±1.09)	7.51 (±1.14)	0.39 (±0.01)	0.22 (±0.04)


### Microbial and Aggregate Counts

**FIGURE 4:**
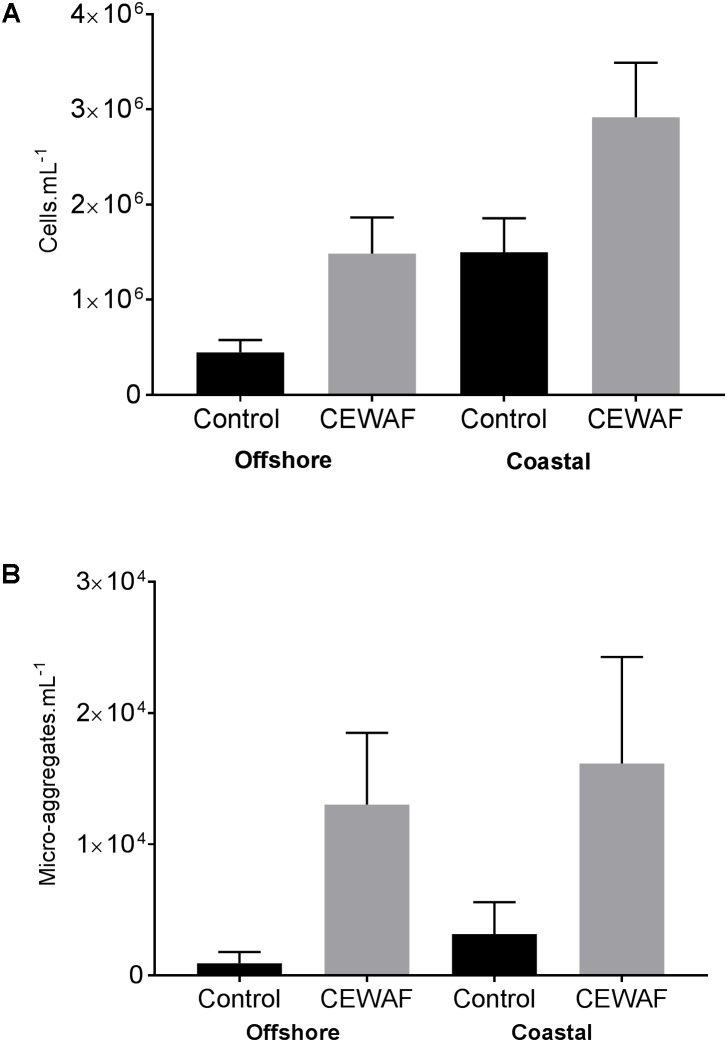
**(A,B)** Average bacterial and micro-aggregate numbers (±SD) in Control and CEWAF treatments from the offshore and coastal mesocosms.

**FIGURE 5:**
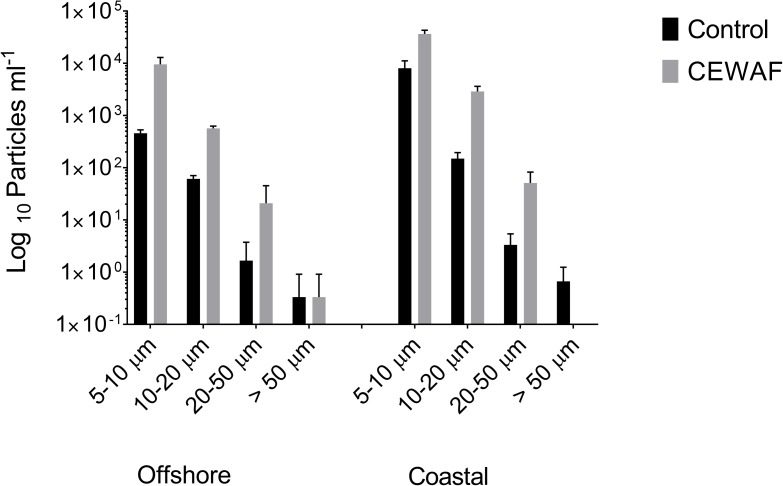
Average log_10_ particle number determined using a coulter counter (±SD) in the size ranges 5–10, 10–20, 20–50, and >50 μm in Control and CEWAF treatment from the offshore and coastal mesocosms.

Compared to the Control, bacterial counts in the CEWAF treatments were approximately 3.3 fold higher in offshore and nearly 2 fold higher in coastal seawater (**Figure 4**). Similarly, the micro-aggregate counts in CEWAF were nearly 14 fold higher in offshore and 12 fold higher in coastal as compared to Control treatments (Two-way ANOVA: *p* < 0.0001). Further analysis of the aggregates based on size showed significantly higher particle concentrations in 5–10, 10–20, and 20–50 μm size range in the CEWAF treatment compared to the Control (Two-way ANOVA: *p* < 0.0001) (**Figure 5**). Control tanks had significantly higher number of particles than CEWAF treatments in the size range of >50 μm in the coastal mesocosm (Two-way ANOVA: *p* < 0.0001); however, no differences were observed for the same size range in offshore tanks. Particles abundance was more similar between coastal and offshore tanks across all the size ranges except for 10–20 μm, where it was higher in the coastal mesocosm (Two-way ANOVA: *p* < 0.0001) (**Figure 5**).

### Microbial Community Composition

**FIGURE 6:**
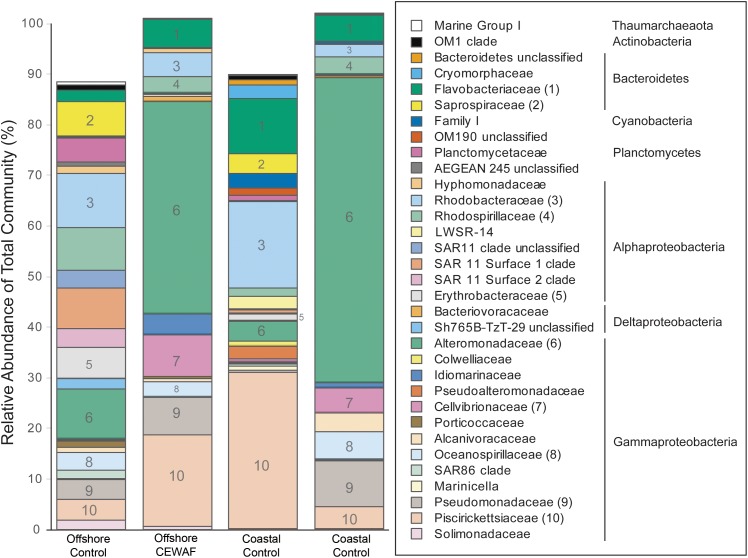
Prokaryotic relative abundance assessed via Illumina sequencing of the V4 region of 16S rRNA. Results are mean of three replicate tanks per treatment. Only taxa representative of >0.5% of the total community in *at least* one sample are shown. The 10 families shown in Figure are indicated by number in the bars and in parentheses after the family name in the key. Phylum and Class (for Proteobacteria) are given for the families identified in the key at right.

Prokaryotic communities in Control treatments were more diverse in both mesocosms than in the CEWAF treatments (Supplementary Figure S2 and Supplementary Table S1). Based on 16S rRNA amplicon sequence data, Bacteria were detected at much higher relative proportion than Archaea in both mesocosm experiments (**Figure 6**). Microbial community composition was dramatically different between the Control and CEWAF samples for both the coastal and offshore mesocosms. The mean relative abundance of the family *Alteromonadaceae* was highest, followed by *Piscirickettsiaceae*, *Rhodobacteraceae*, *Flavobacteriaceae, Pseudomonadaceae*, and *Rhodospirillaceae*. Among these abundant families, the genera with the highest relative abundances was *Marinobacter*, followed by *Alteromonas* (both belonging to the family *Alteromonadaceae*), *Methylophaga* (*Piscirickettsiaceae*), unclassified *Oceanospirillales*, *Cycloclasticus* (*Piscirickettsiaceae*), *Aestuariibacter* (*Alteromonadaceae*) and *Tenacibaculum* (*Flavobacteriaceae*). Three different OTUs of *Marinobacter* (family *Alteromonadaceae*), OTU3, OTU8 and OTU11, represented 21% of the CEWAF community in the offshore and 45% of the CEWAF community in the coastal experiments, respectively (Supplementary Table S2). OTU level responses were not always uniform within a genus. For example, the relative abundance of the two most abundant OTUs of *Methylophaga* (family *Piscirickettsiaceae*) was different between treatments, with OTU2 having highest relative abundance in CEWAF in the offshore mesocosm and OTU5 exhibiting higher relative abundance in the Control for both mesocosm experiments.

**FIGURE 7:**
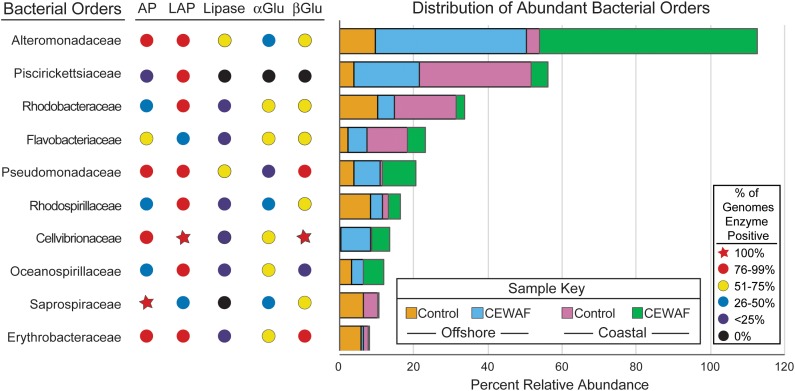
Distribution of bacterial orders with high relative abundance (defined as orders which are present at ≥1% relative abundance in two or more samples) and percentages of sequenced genomes containing exoenzymes assayed. The *x*-axis measures the relative abundance of taxa. AP, alkaline phosphatase; LAP, leucine aminopeptidase; αGlu, α-Glucosidase; βGlu, β-Glucosidase. The sequence of the genes coding for these enzymes were obtained from IMG database.

Compared to all the extracellular enzymes, α-glucosidase was present in the lowest percentage (<75%) of genomes amongst all the abundant bacterial orders (**Figure 7**). β-glucosidase was 100% positive in *Cellvibrionaceae*, members of which were abundant in both the CEWAF treatments. In addition, β-glucosidase was highly positive (76-99%) in *Pseudomonadaceae*, which was abundant in both CEWAF treatments and the offshore Control treatments (**Figure 7**). Lastly, *Erythrobacteraceae*, which had higher relative abundance in the offshore mesocosm than the coastal mesocosm were highly positive for β-glucosidase as well (**Figure 7**). *Alteromonadaceae* and *Pseudomonadaceae* had the highest percentage of genomes positive for lipase (51–75%) (**Figure 7**). Both these orders, especially *Alteromonadaceae*, were significantly abundant in both the CEWAF treatments. Relative to other extracellular enzymes, leucine amino-peptidase was highly positive (76–100%) amongst all the abundant bacterial orders with the exception of *Flavobacteriaceae* and *Saprospiraceae* (**Figure 7**). Alkaline phosphatase was highly positive (76–99%) amongst *Alteromonadaceae*, *Pseudomonadaceae* and *Cellvibrionaceae*, which were abundant in both the CEWAF treatments (**Figure 7**). Amongst bacterial orders abundant in the Control treatments, alkaline phosphatase were 100% positive in *Saprospiraceae* and highly positive in *Erythrobacteraceae* (**Figure 7**).

## Discussion

The goal of this study is to better understand the role of extracellular enzymes, EPS production, and microbial community composition in the presence of a mixture of oil and dispersant. Five different enzymes: α- and β-glucosidase, lipase, alkaline phosphatase and leucine amino-peptidase were studied in the surface, water column and bottom of the mesocosm tanks filled with either coastal or offshore water from the Gulf of Mexico with and without CEWAF. Elevated extracellular enzyme activities have been previously reported in oil containing aggregates (Ziervogel et al., 2012). Also dense microbial colonization and active degradation of oil and components of EPS have been reported (Ziervogel et al., 2012, 2016; Arnosti et al., 2016), suggesting a link between extracellular enzymes and microbial aggregates.

α and β-glucosidase help in the digestion of complex polysaccharides (Reese et al., 1968). These enzymes help in cleavage of glycosidic bonds with α and β-glucosidase acting at α and β -linkages allowing the release of glucose molecule from polysaccharides (de Melo et al., 2006). In the Control treatments, highest α and β-glucosidase activity was seen in layers whereby the polysaccharide concentration in the EPS was lower and vice-versa. Similar patterns but relatively higher levels of activity, were observed in CEWAF treatments as well. This inverse relationship between glucosidase activity and EPS polysaccharide concentration across the three layers is indicative of active breakdown of polysaccharides by these enzymes.

α and β-glucosidase have been shown to help the turnover of polysaccharides secreted by phytoplankton and bacteria, as EPS fueling heterotrophic metabolism in the ocean (Piontek et al., 2010). We therefore hypothesize that the increased activity of these enzymes at the bottom for the Control and surface for CEWAF might help breakdown of the EPS, thereby enhancing the availability of simple carbon in the form of glucose. In addition, compared to the Control, a relatively higher glucosidase activity was observed in CEWAF in both the offshore and coastal mesocosms. Enhanced polysaccharide degradation by extracellular enzymes, especially laminarin, which are targets of glucosidase was observed by Arnosti et al. (2016) in oil associated aggregates. In addition, the polysaccharide concentrations decreased in the Control treatments from ∼0.15 mg.L^-1^ at 0 hrs (data not shown) to ∼0.0003 mg.L^-1^ at 96 h in the bottom of offshore and ∼0.18 mg.L^-1^ (data not shown) to 0.0002 mg.L^-1^ in the coastal mesocosm. Similarly in the CEWAF tanks, the polysaccharide concentrations decreased from ∼0.19 mg.L^-1^ (data not shown) at 0 h to ∼0.0009 mg.L^-1^ at 72 h in the surface of offshore and ∼0.36 mg.L^-1^ (data not shown) to 0.002 mg.L^-1^ for the coastal mesocosm. We therefore hypothesize that CEWAF induced enhanced EPS secretion in the form of polysaccharide at an earlier time point of this experiment that may have been rapidly degraded by these glucosidases.

Ziervogel et al. (2012) previously showed a correlation between lipase activity and oil degradation. Therefore, the higher lipase enzyme activity at the surface and at the bottom of the mesocosm tanks for the CEWAF treatments, along with other hydrocarbon degrading enzymes (not measured in this study), might have played a role in breakdown of the oil components. Analysis of relative prokaryotic abundance showed the presence of lipase producing families, such as *Alteromonadaceae*, *Oceanospirillaceae*, and *Pseudomonadaceae*, in relatively higher numbers. Several other studies focusing on DwH oil spill have reported the presence of these families in samples containing high concentrations of oil (Dubinsky et al., 2013; Lamendella et al., 2014; King et al., 2015; Campeão et al., 2017). Additionally, the lipase producing genomic capabilities detected among members of the families *Altermonadaceae*, and *Pseudomonadaceae* suggest that these taxa might have played a significant role in the degradation of oil in the CEWAF treatment. The products of these extracellular hydrocarbon degrading enzymes might have led to increased carbon availability in the CEWAF treatment.

Leucine aminopeptidase (LAP) helps microbes acquire nitrogen by breaking down proteins and peptide molecules (Fruton and Mycek, 1956). Likewise, alkaline phosphatase is responsible for degradation of organic phosphates, providing a source of phosphorus to microbes (Martínez, 1968). Both leucine amino-peptidase and alkaline phosphatase activity patterns had higher activities at the surface than in the water column and the bottom of the mesocosm tanks for CEWAF treatments. EPS protein content was higher in the water column of the mesocosms than at the surface or bottom, similar to that observed for polysaccharides. Moreover, the higher leucine amino-peptidase and alkaline phosphatase activities at the surface for the CEWAF treatment pattern matches well with α- and β-glucosidase activities. This suggests active assimilation of nitrogen and phosphorus through leucine amino peptidase and alkaline phosphatase along with carbon through α and β-glucosidase from the EPS, which could have contributed in the generation of microbial biomass. There are other reports where enzyme activities have been shown to correlate with the C:N:P requirements of microbes (Sinsabaugh et al., 2008, 2009). Therefore, we hypothesize that the products of these extracellular enzyme activities observed in our mesocosm tanks may have supported microbial requirements that lead to increased microbial biomass observed in the CEWAF tanks.

Analysis of alkaline phosphatase, leucine amino-peptidase and β-glucosidase in whole water and <10 μm size fractions were statistically indistinguishable (Whitaker, personal communication), indicating that at least large eukaryotes > 10 μm are not responsible for the vast majority of activity measured. These assumptions aside, similar to other studies (Gutierrez et al., 2013; Kleindienst et al., 2015b; Yang et al., 2016a,b), our observations showed that *Alteromonadaceae*, represented largely by *Marinobacter*, *Alteromonas*, and *Aestuariibacter*, are the most abundant bacterial order detected and also very commonly have alkaline phosphatase and leucine aminopeptidase. This indicates a potentially large contribution to overall enzyme activity from these genera to alkaline phosphatase and leucine aminopeptidase enzyme activity rates. In particular, for AP and Lipase, the percentages of enzyme positive genomes are highly uneven, indicating that a consortia of microbes is necessary to efficiently hydrolyze EPS *in situ*.

It is also interesting that the family *Piscirickettsiaceae* is abundant but seems to only use leucine amino-peptidase out of the five enzymes in any significant proportion. All other abundant prokaryotes potentially contribute to 3–4 of the measured enzymes in a similar proportion within the family, but *Piscirickettsiaceae* operate outside this trend. This may indicate that this abundant family gets its phosphorus and carbon through other means. The two genera common in our mesocosm experiments, *Methylophaga* and *Cycloclasticus*, are putative hydrocarbon degraders (Gutierrez and Aitken, 2014). Therefore, they may have used hydrocarbons for carbon and enzymes other than lipase or glucosidases to metabolize that carbon-source.

Some members of the most abundant families in our mesocosms are known as “cheaters”; abundant community members that have minimal to no contribution in terms of enzyme activity (Allison, 2005). For example, members of the family *Planctomycetaceae* and SAR11 clades (Unclassified, Surface 1, and Surface 2) were abundant in the Control treatments but genomes from these lineages do not harbor abundant exoenzymes: < 10% of *Pelagibactereaceae* genomes have any of the enzymes assayed here, with the exception of leucine aminopeptidase, which is present in 47% of the genomes queried. Interestingly, there were almost no families lacking extracellular enzyme producing ability (cheaters) in the CEWAF treatments. This could be due to the potential toxicity of oil and Corexit, or the hypoxic environment in the CEWAF treatments restraining the microbial community exclusively to a few dominant families that are being selected due to their ability to tolerate and degrade hydrocarbons (Hamdan and Fulmer, 2011; Bacosa et al., 2012, 2015b; Liu et al., 2017). Such reduced microbial diversity limited to fewer families such as *Alteromonadaceae*, *Pseudomonadaceae*, *Oceanospirillaceae*, *Piscirickettsiaceae*, and *Idiomarinaceae* has been reported in other studies focusing of DwH oil spill (Kleindienst et al., 2015b; Yang et al., 2016a,b).

The overall abundance of potential oil degraders such as members of *Alteromonadaceae*, *Pseudomonadaceae*, *Cellvibrionaceae*, and *Piscirickettsiaceae* in the CEWAF treatments suggests oil may have acted as the primary carbon source. However, since the glucosidase were higher in CEWAF treatments, we hypothesize the products of glucosidases may have provided additional carbon source as well. Moreover, the heavy supply of carbon also increases the demand for other elements such as nitrogen and phosphorus, which are not present in oil. We assume, some portions (in addition to the supplemented nutrients in the mesocosm tanks) of the required high levels of nitrogen and phosphorus to support the growth may have been provided by means of extracellular enzyme reactions such as alkaline phosphatase and leucine amino-peptidase. The overall activities of enzymes were relatively higher in CEWAF treatments than Control, and similar observations were made by Kleindienst et al. (2015b). Therefore, the products of these enzymes may have played a role in the high cell numbers seen in this treatment relative to Control. Several reports indeed have shown that the products of these extracellular enzyme can support the growth of bacteria (Frankenberger and Dick, 1983; Vetter and Deming, 1999). Apart from higher cell counts, statistically higher micro-aggregate counts was also observed in the CEWAF treatments compared to Control. This can be explained by relatively higher protein content of the EPS produced in response to CEWAF. We hypothesize that increase in proteins content may have enhanced the amphiphilic nature of the EPS, which may have facilitated better interaction of the EPS with the oil. This in-turn may lead to formation of more micro-aggregates in CEWAF treatment. Such role of EPS interaction with oil in the formation of aggregates has been previously suggested (Passow et al., 2012; Kleindienst et al., 2016; Quigg et al., 2016). Although, the enzyme activities and EPS production were higher in the CEWAF treatments than in Control, any interpretation on the effect of dispersant addition alone has to be taken with caution as the study did not compare CEWAF to WAF or a Corexit only treatment. Kleindienst et al. (2015b) suggested Corexit could suppress microbial activity, on the other hand, Bacosa et al. (2015a,b) showed positive effect on growth and alkane degradation in the presence of the dispersant Corexit. There were many differences between the experimental conditions used in Bacosa et al. (2015a,b) and Kleindienst et al. (2015b) such as temperature (8°C vs. ambient temperature similar to our study) and site of sampling depth (1500 m vs. surface similar to our study). Kleindienst et al. (2015b) worked with a closed bottle system in the dark for 6 weeks while our study lasted a few days in an open system. These differences and others in experimental conditions may have influenced the outcome of dispersant addition on microbial activity. Therefore, despite the observation of higher microbial activity in our CEWAF treatments, further studies are essential to gain a more detailed understanding the effects of dispersant addition alone. Different levels of enzyme activities, EPS production, cell and aggregate counts were observed in response CEWAF of the offshore relative the coastal mesocosm. We hypothesize that these differences could be primarily due to the different initial conditions in the offshore and coastal waters.

## Conclusion

Our study shows that addition of oil and dispersant Corexit enhances extracellular enzyme activity and EPS production in relative to Control, and a similar comparison between oil and dispersant mixture with oil only treatment is needed. Microbial community in CEWAF treatment was mostly dominated by hydrocarbon degraders such as members of *Alteromonadaceae*, *Pseudomonadaceae* and *Cellvibrionaceae*. The higher protein content of EPS in response to CEWAF may have facilitated increased aggregation. However, further studies comparing CEWAF treatment with WAF with time course measurements are needed to discern the effect of dispersant addition on extracellular enzyme and EPS production and aggregation.

## Author Contributions

MK designed the experiment, conducted the enzyme measurements and data analysis, and wrote the manuscript. CX helped in conducting the EPS and carbon analysis and manuscript preparation. KS helped in designing the experiment and writing of the manuscript. LB helped in measurements of the particle size and manuscript preparation. MB helped in conducting the EPS and carbon analysis. SD conducted the genomic and associated data analysis and manuscript preparation. JG and JH helped in conducting the experiment. JS helped in genomic data analysis and manuscript preparation. PS helped in designing the experiment and manuscript preparation. AQ mentored the study and helped in designing the experiment and manuscript preparation.

## Conflict of Interest Statement

The authors declare that the research was conducted in the absence of any commercial or financial relationships that could be construed as a potential conflict of interest.
