# Supplementary material for: Extracellular Enzyme Activity Profile in a Chemically Enhanced Water Accommodated Fraction of Surrogate Oil: Toward Understanding Microbial Activities After the Deepwater Horizon Oil Spill
Source: Front Microbiol. 2018 Apr 24;9:798. doi: 10.3389/fmicb.2018.00798 (PMC5928240; doi:10.3389/fmicb.2018.00798)
Supplement: Supplementary file 1 [file Table_1.DOCX]

**Table S1.** Comparison of alpha diversity metrics observed in the samples used for this study using 16S rRNA hyper-variable V4 region sequence reads clustered with a 3% dissimilarity cutoff. Data presented is mean for three replicate tanks.

| **Sample** | **# of Reads (subsampled)** | **Good's Coverage** | **OTUs observed** | **Chao1** | **Inverse Simpson** |
| --- | --- | --- | --- | --- | --- |
| M3_T8_Control | 39,054 | 0.996 | 444 | 680 | 19.1 |
| M3_T8_CEWAF | 39,054 | 0.997 | 327 | 523 | 13.9 |
| M4_T6_Control | 39,054 | 0.994 | 639 | 903 | 28.6 |
| M4_T6_CEWAF | 39,054 | 0.995 | 530 | 764 | 23.7 |
