# Supplementary material for: Extracellular Enzyme Activity Profile in a Chemically Enhanced Water Accommodated Fraction of Surrogate Oil: Toward Understanding Microbial Activities After the Deepwater Horizon Oil Spill
Source: Front Microbiol. 2018 Apr 24;9:798. doi: 10.3389/fmicb.2018.00798 (PMC5928240; doi:10.3389/fmicb.2018.00798)

Figure S1. Average total enzyme activities (± SD) in Control and CEWAF treatments from the offshore and coastal mesocosms.

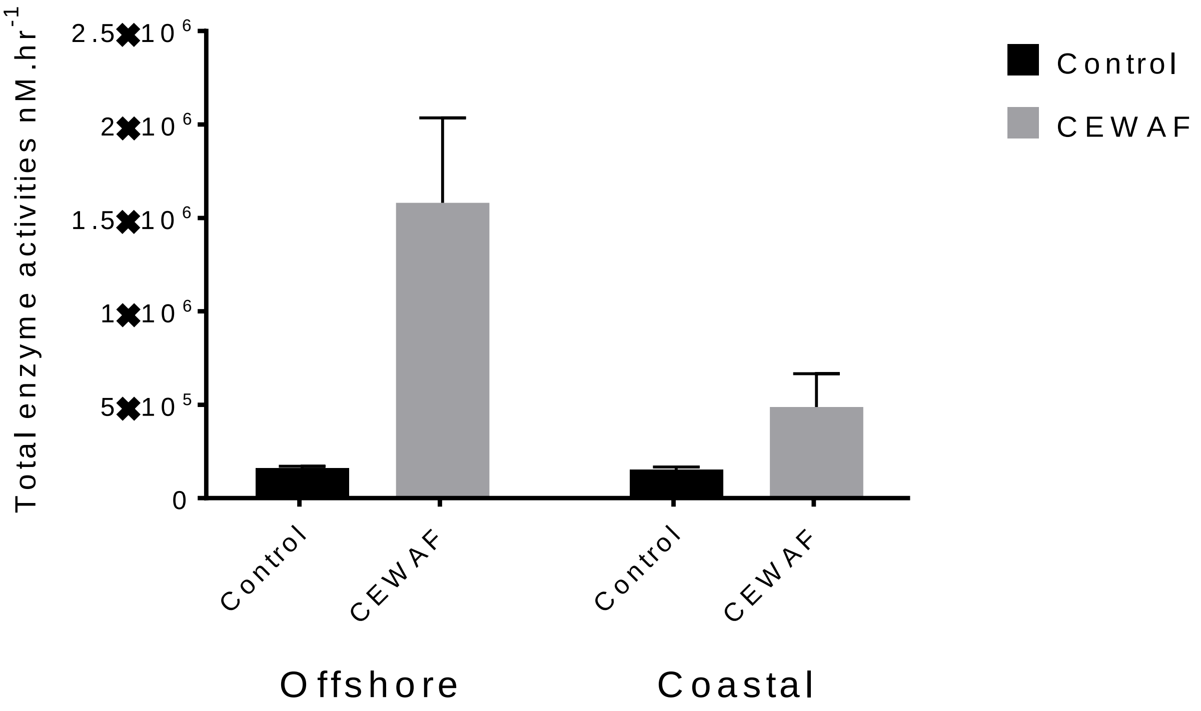

Figure S2

Rarefaction analysis of 16S rRNA libraries from the four treatments. Grey lines are for control samples, blue lines for CEWAF. Data presented is mean for three replicate tanks.

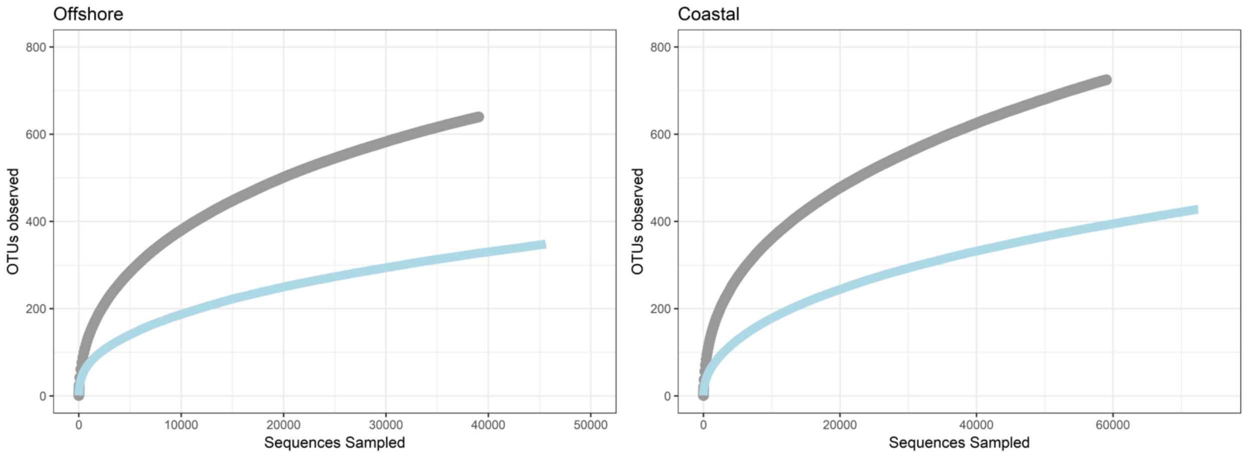

Supplement: Supplementary file 3 [file Image_1.PDF]
